# Anatomical Aspects of Neurogenic Bladder and the Approach in Its Management: A Narrative Review

**DOI:** 10.7759/cureus.31165

**Published:** 2022-11-06

**Authors:** Samayak J Kumar, Dalia A Biswas

**Affiliations:** 1 Urology, Jawaharlal Nehru Medical College, Datta Meghe Institute of Medical Sciences, Wardha, IND; 2 Physiology, Jawaharlal Nehru Medical College, Datta Meghe Institute of Medical Sciences, Wardha, IND

**Keywords:** autonomous bladder, atonic bladder, cortical bladder, spastic bladder, onuf's nucleus, pontine micturition centre, neurogenic bladder

## Abstract

The contraction of the detrusor muscle causes the urinary bladder and its mass peristaltic movement, leading to micturition. The vesical plexus of nerves, composed of fibers from the inferior hypogastric plexus, supplies the urinary bladder. The brain plays a crucial part in developing and maintaining bladder control, although its specific involvement in urgency and urine leakage is not well understood. The critical components in the neural control of the bladder and its regulation are the pontine micturition center (located in the mediodorsal aspect of the pons) and the Onuf's nucleus, also known as the sacral micturition center (located between the sacral S2 and S4 segments). The most important cause of a neurogenic bladder is damage or lesions of the spinal cord affecting the pontine micturition center, Onuf's nucleus, or damage to the motor neurons between the pontine and the sacral centers of micturition. Neurogenic bladder can be of several types based on the location of the lesions, such as the autonomous bladder, spastic bladder, atonic bladder, and cortical bladder, all were presented with a unique clinical picture. The classical approach to a case of neurogenic bladder involves a complete assessment of the neurologic system and of pelvic anatomy, while neurogenic bladder rehabilitation may include a bladder retraining program involving intermittent catheterization, timed voiding, medications, and lifestyle modifications. This review article attempts to correlate the neurogenic bladder with various anatomical aspects related to the micturition center in the brain and spinal cord and their control over the urinary bladder, as well as the classical approach toward such a case of neurogenic bladder.

## Introduction and background

The urinary bladder stores urine until the micturition reflex is triggered. It is included within the anterior pelvic compartment. The mass peristaltic movement of the bladder, which leads to micturition, is caused by the contraction of the detrusor muscle, the smooth muscle of the urinary bladder, whose muscle fibers extend in all directions, which, when contracted, can increase the pressure in the bladder up to 40 to 60 mm Hg. The urinary bladder is supplied by the vesical plexus of nerves, which is made up of fibers generated by the inferior hypogastric plexus. Each of the sympathetic and parasympathetic components of the vesical plexus comprises both motor and sensory fibers [1,2]. Parasympathetic efferent nerve fibers control the detrusor muscle (S2 to S4) and do not supply the pre-prostatic sphincter. If they are lost, regular micturition cannot occur. It is believed that sympathetic efferent fibers (T11 to L2) are inhibitory to the detrusor and motor to the pre-prostatic sphincter mechanism. The voluntary sphincter urethra is placed in the urethra's wall and innervated by the somatic pudendal nerve (S2-4).

The parasympathetic and sympathetic nerves carry pain sensations generated by bladder distension or spasm, respectively. In the spinal cord, bladder pain is mediated by the lateral spinothalamic tract, while bladder distension is felt via the posterior columns. Therefore, bilateral anterolateral cordotomy reduces pain selectively without changing the impression of bladder distension or the urge to urinate. Within the peripheral nervous system, preganglionic axons exhibit significant divergence to form synapses with many ganglionic targets. In all ganglia, synaptic transmission is mediated by acetylcholine (Ach) acting on nicotine receptors [3]. One of the most critical steps in micturition is contraction of the detrusor muscle. "The external sphincter muscle is under the voluntary control of the nervous system and can be used to consciously prevent urination, even when involuntary controls are attempting to empty the bladder" [4]. Disorders leading to the impairment or malfunctioning of the micturition reflex, which subsequently leads to urinary incontinence, may lead to various adverse psychosocial outcomes [5]. Multiple studies reported the feeling of powerlessness and impairment of engagement in daily activities and social participation.

On literature search, it has been found that there is a persistent dilemma about the control of the bladder by the brain as well as the spinal cord. This review article highlights the straightforward understanding of bladder control by the micturition center in the brain as well as the spinal cord and its correlation with the sympathetic and parasympathetic nervous systems. The present review also discusses various types of neurogenic bladders and their anatomical basis.

Search methodologies

**Figure 1:**
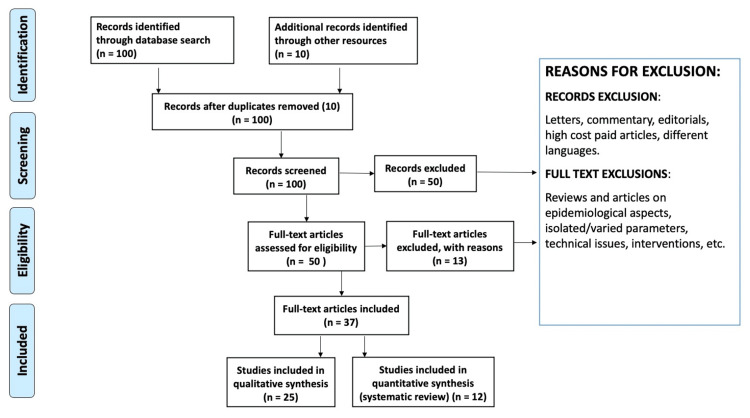
Review of literature using the PRISMA method PRISMA, Preferred Reporting Items for Systematic Reviews and Meta-Analyses

We conducted extensive research to discover the literature using PubMed, Medline, Cochrane Library, and Web of Science and utilized the following keywords to sensitize or search: pontine micturition center, neurogenic bladder, Onuf’s nucleus, spastic bladder, atonic bladder, cortical bladder, autonomous bladder. All literature was screened for appropriateness by title. We excluded articles that were incomplete, not in the English language, or duplicate. We included articles showing the anatomical aspects of the neurogenic bladder. First, we used titles and abstracts to screen the identified studies. A second selection process was applied to the records determined to be acceptable at this stage based on the full-text examination. Each time a decision needed to be made, the researchers explored potential differences in the study choice until they came to an agreement. Figure 1 shows the flow of study selection according to PRISMA (Preferred Reporting Items for Systematic Reviews and Meta-Analyses) method of literature search. The following data was collected for each study: name of the first author, year of publication, research population, age, sex, the definition of neurogenic bladder, outcome, key findings, and conclusion.

## Review

Neural regulation and micturition reflex

Since information about the urinary bladder filling is transmitted from the spinal cord to the brainstem and back to the spinal cord, the micturition reflex is frequently referred to as a spinobulbospinal system [6]. The pontine micturition center (PMC) is located in the brain stem and is responsible for the coordination of relaxation of the external sphincter to synchronize with bladder contractions [7]. The micturition reflex, whose reflex center is located in the rostral pontine tegmentum, is a bladder-to-bladder contraction reflex. There are two afferent circuits from the bladder to the brain. The first is the dorsal system, whereas the second is the spinothalamic tract [8]. When the PMC is active, its primary function is to promote urination. It accomplishes this by causing the detrusor muscle to contract and indirectly inhibiting the somatic nerves that keep the external sphincter contracted by the activation of parasympathetic neurons [9]. In other words, PMC acts as an on-off switch that is signalled by stretch receptors in the bladder wall, which, in turn, is modulated by inhibitory and excitatory influences from the brain. The sacral micturition center governs bladder contraction and is positioned between the sacral S2 and S4 vertebrae. This region is a reflex center where afferent impulses from the bladder indicate bladder fullness and efferent parasympathetic impulses to the bladder cause bladder contraction [7].

**Figure 2:**
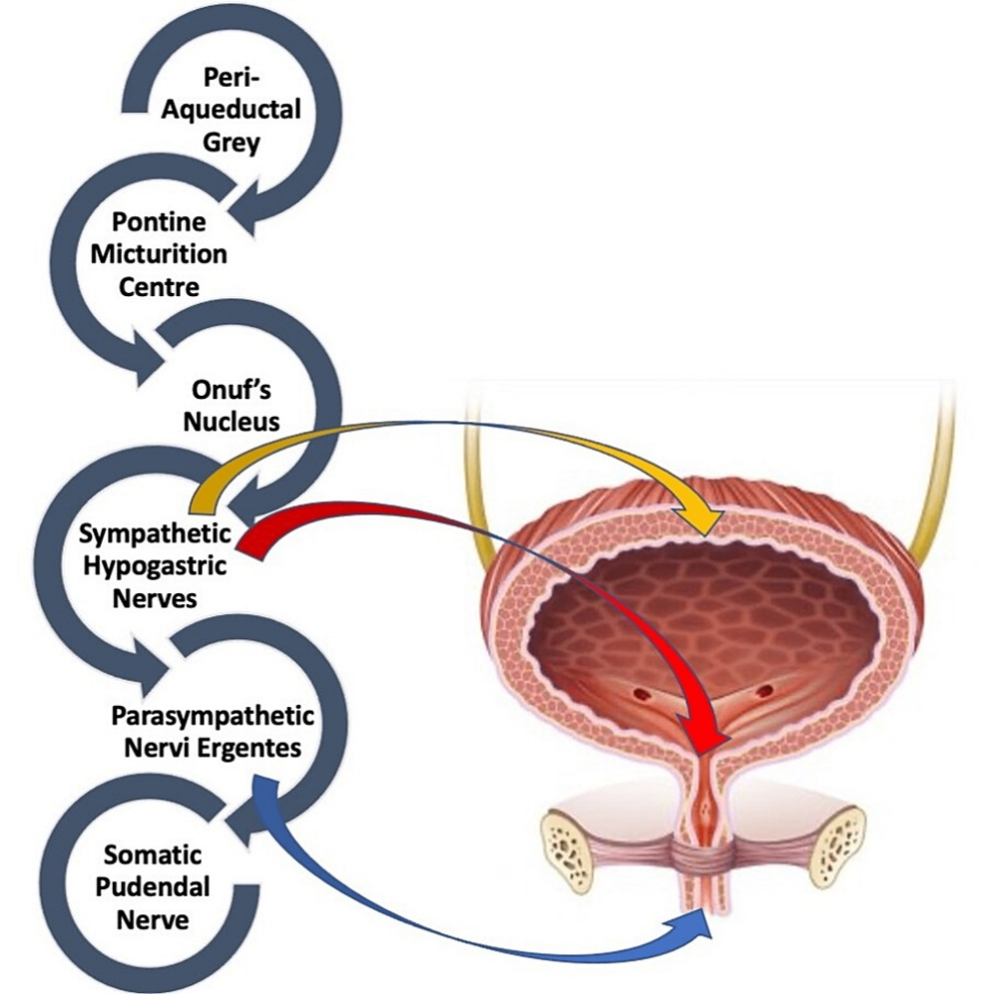
Correlation of coordination between the nervous system and bladder control

The micturition reflex and urinary continence are both significantly influenced by Onuf's nucleus. These neurons are located on the lateral side of the ventral horn of S2-S4 [10,11]. Neurons in this nucleus receive cortical inputs and noradrenergic and serotonergic facilitatory inputs via interneurons from numerous brain stem regions, including the pontine urine storage center [12,13]. The central role of Onuf’s nucleus is in the control of the rhabdosphincter [14], responsible for conscious inhibition of the micturition reflex [10,12]. “Once the micturition reflex becomes powerful enough, it causes another reflex, which passes through the pudendal nerves to the external sphincter to inhibit it. If this inhibition is more potent in the brain than the voluntary constrictor signals to the external sphincter, urination will occur. If not, urination will not occur until the bladder fills further, and the micturition reflex becomes more powerful "[3]. A diagrammatic representation of the control of various areas of the central nervous system (CNS) over bladder musculature is shown in Figure 2.

The classification of neurogenic bladder dysfunction is based on the location of neurologic lesions, leading to clinically different types of voiding abnormalities, and this guides appropriate surgical and pharmacological therapy.

Neurogenic bladder and its correlation with the CNS

Neurogenic bladder refers to a group of disorders occurring due to damage or various diseases of the CNS [7,10]. A neurogenic bladder can arise due to lesions or trauma to the PMC (e.g., brain tumor or stroke), trauma to motor neurons between the PMC and sacral center (Onuf’s nucleus), spinal cord injury, or demyelinating diseases such as multiple sclerosis. This leads to upper motor neuron bladder (UMNB) or spastic bladder. Peripheral nerve pathology such as diabetic neuropathy can lead to neurogenic bladder dysfunction due to autonomic and peripheral neuropathy [15]. Various types of neurogenic bladders with etiopathogenesis and clinical features have been highlighted in Table 1.

**Table 1:** Types of neurogenic bladders and their etiopathogenesis and salient clinical features PMC, pontine micturition center; LMNB, lower motor neuron bladder

Sr. no.	Types of bladder and its pathogenesis	Site of lesion	Etiology	Clinical manifestations
1a	Detrusor areflexia (temporary acute cerebral shock phase)	Supraspinal lesions	Lesion in the central nervous system above the pons. Causes are cerebrovascular episodes, brain tumor, Parkinson disease, and Shy-Drager syndrome	Urinary retention
1b	Detrusor hyperreflexia with coordinated urethral sphincter activity (PMC is released from the cerebral inhibitory center)			Urinary frequency, urinary urgency, and urge incontinence
2a	Detrusor areflexia in spinal shock stage (somatic reflex activity and autonomic activity either depressed or absent)	Spinal lesions	Spinal cord trauma	Urinary retention
2b	Detrusor hyperreflexia after the spinal shock			
2c	Detrusor sphincter dyssynergia–detrusor hyperreflexia (depending on the level of lesion)			
3a	Detrusor hyperreflexia, striated sphincter dyssynergia, smooth sphincter dyssynergia, and autonomic dysreflexia	Spinal cord lesions above the T6 vertebra	Spinal cord lesions and demyelinating diseases such as multiple sclerosis	Sweating, headache, hypertension, and reflex bradycardia
3b	Detrusor hyperreflexia, striated sphincter dyssynergia, and smooth sphincter dyssynergia but no autonomic dysreflexia	Spinal cord lesions below the T6 vertebra		Neurologic examination of UMN-spasticity, hyperreflexic deep tendon reflexes, and extensor plantar response
4	Detrusor areflexia (autonomic and peripheral neuropathy)	Peripheral nerve lesions	Diabetes mellitus, Tabes dorsalis (neurosyphilis), herpes zoster, herniated lumbar disc disease	Loss of sensation of bladder filling followed by loss of motor function, decreased bladder sensation, impaired detrusor contractility (LMNB)

Damage to the spinal cord at the sacral level that damages the detrusor nucleus but spares the pudendal nucleus leads to a mixed type A bladder, damage to the spinal cord at the sacral level that spares the detrusor nucleus but at the same time damages the pudendal nucleus leads to a mixed type B bladder, and trauma to the sacral cord or the sacral nerve roots (damage to Onuf’s nucleus) leads to lower motor neuron bladder (LMNB).

Autonomous Bladder

Detrusor overactivity is a result of the autonomous micromotility of the detrusor muscle in the storage phase. This may be due to local denervation due to neurogenic, obstructive, or idiopathic overactivity [16,17]. Urge incontinence is one of the most common symptoms of an overactive bladder [18]. Studies have shown that an overactive bladder may develop in a variety of patients, including the elderly, men with prostatic enlargement, or post-menopausal women [19,20].

Detrusor underactivity is defined by a prolonged duration of urination with or without a sensation of incomplete bladder emptying, typically accompanied by hesitancy, diminished sensation upon filling, and a slow stream [21]. This could be the result of similar local denervation but a greater extent of damage, leading to a low contraction response.

Spastic Bladder

Also known as UMNB, this condition is caused by traumatic damage to efferent sympathetic fibers or multiple sclerosis of the cervicothoracic spinal cord. The spastic bladder is marked by detrusor-sphincter dyssynergia, in which simultaneous detrusor and urinary sphincter contractions produce high pressures in the bladder (up to 80-100 cm H_2_O), resulting in vesicoureteral reflux, which can cause renal damage [22]. The bladder and the sphincter both become spastic due to spinal cord injury particularly localized above the level of the T10 vertebra (above the sympathetic autonomic nervous system innervation of the bladder). Neurogenic detrusor overactivity or detrusor hyperreflexia is associated with decreased bladder capacity.

In the mixed type A neurogenic bladder, which has a higher incidence rate among mixed neurogenic bladders, destruction of the detrusor nucleus leads to detrusor paralysis (detrusor areflexia), while the undamaged pudendal nucleus is spastic, leading to a hypertonic outer rhabdosphincter. Due to the enormous size and low pressure inside the bladder, the spastic external sphincter causes urine retention.

The mixed type B neurogenic bladder is characterized by a dysfunctional outer urinary sphincter caused by a lesion to the pudendal nucleus and a spastic bladder caused by a detrusor nucleus that has uninhibited regulation. Consequently, the bladder capacity is minimal, although pressure inside the bladder is typically not increased due to the low outflow resistance. However, this causes issues with incontinence. Due to the elevated detrusor tone, the maximum volume of the bladder is typically diminished (neurogenic detrusor overactivity or detrusor hyperreflexia).

Atonic Bladder

It involves damage to the lower sacral centers (damage to Onuf’s nucleus) and is hence also known as the LMNB. The bladder capacity may be raised in an atonic bladder due to the intact innervation of the internal urinary sphincter and the low detrusor tone (detrusor areflexia). Urinary overflow incontinence and urinary tract infections are prevalent despite the decreased detrusor pressure [23].

Important causes of the atonic bladder are spina bifida [24], diabetic neuropathy, multiple sclerosis, traumatic injuries, long or difficult vaginal childbirth, pelvic surgery, enlarged prostate, pelvic tumor, and urethral stricture.

Cortical Bladder

In order to modulate voiding, a network of brain regions is activated throughout the cortical area during voiding [25]. The para-central lobule includes the major cerebrum cortical regions for voiding and storing [26]. Activation of the left thalamus, middle frontal gyrus, superior frontal gyrus, superior precentral gyrus, and the caudal region of the anterior cingulate gyrus during normal micturition was reported in a positron emission tomography (PET) scan [27]. The mid-cingulate cortex on both sides of the brain showed increased activity concerning rising bladder volume. In contrast, the mid-cingulate gyrus on both sides of the brain showed decreased activity in relation to reducing the urge to void.

The classic clinical picture of a patient with frontal lobe incontinence is acute urgency and frequency of micturition with urge incontinence in a patient who is socially conscious and disturbed by the incontinence. In such patients, micturition coordination is regular, indicating that the problem lies in central control.

Approach to a case of neurogenic bladder

The approach to a case of neurogenic bladder should initially start with thorough patient history, including a history of genitourinary surgeries and conditions, and history and complaints related to micturition such as dysuria, hesitancy, nocturia, incontinence, and recurrent infections. Sedative/hypnotic, antidepressant, antipsychotic, antihistamine, anticholinergic, antispasmodic, opiate, alpha-adrenergic agonists/antagonists, and calcium channel-blocking medications may play a role in voiding function [22]. It is essential to keep a record of the fluid intake, voiding issues, and voiding patterns. This helps in diagnosing the cause and type of neurogenic bladder and choosing an appropriate treatment plan.

The physical examination must comprise an assessment of the neurologic system and of the pelvic anatomy. The neurological examination is done to determine if any neurological issues are present that may be contributing to the voiding disorder (particularly the sacral dermatomes). There may be mechanical causes, such as prostate enlargement or prolapse of the bladder, which require examination of the urinary system. For rehabilitation of the neurogenic bladder, the CNS examination includes an assessment of the patient's cognition. Other variables that need to be assessed are sexuality, along with the parameters such as hand strength and coordination, joint contractures, and mobility. When the cause is spinal cord injury, a nervous system examination is done to find out the motor level of the lesion, severity of the injury, tone of extremities, rectal sensation or tone, voluntary rectal tone, and bulbocavernosus reflex. One of the most critical investigations includes the measurement of post-void residual (PVR) urine volume by transurethral catheterization, which is used to measure residual urine volume immediately after urination. This technique can be used to estimate the bladder’s capacity to empty entirely in patients with a neurogenic bladder. The PVR urine volume should always be determined after the discontinuation of Foley catheterization or prior to the initiation of the bladder retraining program, which includes intermittent catheterization as its vital component [28]. PVRs constitute an essential step in the prevention of bladder distension and determining the frequency of catheterization. This step is required to maintain the residual urine volume below 400 cc. Below 100 cc, residual urine volumes are likely to be associated with a lesser risk of the development of bacterial cystitis. PVR urine volumes can also be determined by ultrasound, which is a non-invasive procedure when an accurate measurement is not essential. Patients with neurogenic bladder might undergo a 24-hour urine-creatinine cycle to assess and progressively monitor renal function with the use of 125 I-iothalamate as a brief renal clearance test to evaluate the glomerular filtration rate [29]. A urodynamic examination should be performed to assess urinary function. Urodynamic tests are the most conclusive and objective method for identifying anomalies in the bladder and urethra throughout the filling/storage and voiding phases in neurogenic bladder dysfunction [30].

The urinary flow rate is defined as the volume of urine voided per unit of time. This is a non-invasive procedure for quantifying urinary flow, which is based on the strength of detrusor contraction and urethral resistance. A graph plotted for normal urine flow is typically bell-shaped, consisting of a rapid rise to peak flow, a brief peak flow duration, and a rapid decline in urine flow. An increase in urine flow rate may be an indication of detrusor hyperactivity, whereas a decreased flow may be indicative of urinary outlet obstruction or a weak detrusor. Bladder cystometrogram examines bladder compliance and volume, as well as the presence or absence of unrestrained bladder activity [31]. A transurethral catheter linked to a pressure transducer is used to monitor bladder pressures during emptying and filling along with intra-abdominal pressure. It is also important to note the sensation of bladder filling (usually occurring between 100 and 200 cc), the first urge to urinate (generally occurring between 300 and 400 cc), and the strong need to urinate (mostly occurring between 400 and 500 cc), as bladder capacity ranges from 300 to 600 mL [29,31]. Electromyography of the sphincter muscles is performed to see the presence of detrusor sphincter dyssynergia and to find out if voiding is discoordinated because of this issue.

The bladder leak point pressure is the maximal detrusor pressure recorded during passive filling just before the occurrence of urine leakage. Sustained high detrusor pressures can develop in neurogenic bladders with poor compliance, and leak point pressures exceeding 40 cm H_2_O increase the risk of injury to the upper urinary tract [32,33]. Measurement of the urethral outflow resistance is given by the urethral pressure profile (UPP). Several methods have been documented [34,35], but the most common procedure involves the use of a water-filled urethral catheter, which, in turn, is connected to a pressure transducer [36]. This setup is used to monitor urethral pressures. UPP is a standard procedure after cystometry. Some clinical applications of UPP include the detection of urethral instability and stress urinary incontinence; however, its utility in the detection and treatment of a case of neurogenic bladder is less clear [37].

Management of neurogenic bladder

Management of a neurogenic bladder case requires patient education and interventions such as manual expression, timed voiding, medicines, intermittent catheterizations, and indwelling urinary catheters, as well as surgical procedures [38,39]. A technique known as triggered reflex voiding involves stimulation of detrusor muscles by an externally elicited sacral reflex such as scratching the upper medial aspect of the thigh, suprapubic tapping or jabbing, or perineal manipulation and may be used to allow voiding.

The main objectives in the treatment of neurogenic bladder can be summarized as preventing the development of a high-pressure detrusor that can cause upper urinary tract damage, achieving and maintaining continence in order to avoid the consequences of incontinence such as skin maceration and decubitus, minimizing the risk of urinary tract infections (which may lead to further increase in incontinence, decreased bladder capacity and/or autonomic dysregulation) [40], and preventing the development of a high-pressure detrusor, which can lead to upper urinary tract damage [41]. Various study observations related to neurological bladder dysfunctions are highlighted in Table 2.

**Table 2:** Study observations by different authors pertaining to mechanism of neurologic control of bladder function and its relevance in diseased conditions PAG, periaqueductal grey; PMC, pontine micturition center

Sr. no.	Authors and year	Title	Study observations
1.	Sugaya et al., 2005 [8]	Central nervous control of micturition and urine storage	The control of the micturition reflex by various inhibitory mechanisms proves that the nervous system essentially consists of reflexes and their inhibitory mechanisms and that the function of urine storage is a more important aspect compared to micturition amongst bladder functions.
2.	Dorsher and McIntosh, 2012 [22]	Neurogenic bladder	During bladder emptying, the inhibitory outflow of the supraspinal centers to the pontine micturition center is suppressed, reducing thoracic sympathetic outflow and increasing sacral parasympathetic outflow to the lower urinary tract. Detrusor smooth muscle contraction, bladder neck smooth muscle relaxation, and external urinary sphincter skeletal muscle relaxation are the key components in normal bladder physiology and allow the evacuation of urine stored in the bladder.
3.	Ginsberg, 2013 [7]	The epidemiology and pathophysiology of neurogenic bladder	A better understanding of the relationship between neurologic disorders and neurogenic bladder, targeted use of urodynamics, and a comprehensive neurourologic evaluation may help clinicians better manage neurogenic bladder, its symptoms, and complications, and ultimately improve patient outcomes and quality of life.
4.	de Groat et al., 2015 [3]	Neural control of the lower urinary tract	A complex neural network, located at various levels of the peripheral and central nervous system, controls the storage and periodic release of urine by coordinating the activity of smooth and striated muscles of the bladder and urethra. A switch-like activation of reflex voiding is mediated by a spinobulbospinal pathway that is relayed by PAG-PMC circuitry.
5.	Tudor et al., 2016 [39]	Neurogenic lower urinary tract dysfunction: evaluation and management	Lower urinary tract dysfunction is a common complication of neurological disease. Bladder storage and voiding dysfunction depend on the level of the neurological lesion and its evaluation includes parameters such as history taking, urological examination, bladder diary, ultrasonography, and urodynamic testing.
6.	Aizawa and Igawa, 2017 [21]	Pathophysiology of the underactive bladder	Underactive bladder/detrusor underactivity pathophysiology includes detrusor muscle contractility failure, bladder efferent and/or afferent nerve dysfunction, and the failure of CNS coordination in voiding mechanisms.
7.	Lee et al., 2021 [10]	Sophisticated regulation of micturition: review of basic neurourology	Micturition is regulated by the cortex through pathways projecting from the prefrontal cortex and insular cortex to the pontine micturition center. A normally functioning urethral sphincter is found in patients with lesions in the basal ganglia or thalamus. In these patients, when there is voluntary contraction of the rhabdosphincter and the occurrence of noninhibitory detrusor contractions, abnormal micturition reflexes may either decrease significantly or disappear.
8.	This review	Anatomical aspects of neurogenic bladder and the approach in its management: A narrative review	Pontine micturition center in the brain and Onuf’s nucleus in the spinal cord as well as sympathetic and parasympathetic regulatory nervous systems controls the micturition. Focus has also been on the evaluation and understanding of neurologic disorders and neurogenic bladder for the better management of complications hence improvement in patient outcomes and quality of life.

## Conclusions

Micturition is governed by several micturition reflexes, which are under the control of the PMC in the brain and Onuf’s nucleus in the spinal cord. This micturition is also under the significant control of the sympathetic and parasympathetic regulatory nervous systems. The micturition reflex is controlled by several inhibitory mechanisms, which confirms that the nervous system consists of reflexes and their inhibitory mechanisms and that the function of urine storage is more important than micturition among bladder functions. Chronically high detrusor pressures can cause renal damage, and neurogenic bladder dysfunction can be successfully treated to ensure urine continence, avoid renal impairment, and reduce the risk of urinary tract infections or bladder overdistension.
